# Sudden cardiac death after heart transplantation: a population-based study

**DOI:** 10.1093/europace/euad126

**Published:** 2023-05-19

**Authors:** Guillaume Bonnet, Guillaume Coutance, Olivier Aubert, Victor Waldmann, Marc Raynaud, Anouk Asselin, Marie-Cécile Bories, Romain Guillemain, Patrick Bruneval, Shaida Varnous, Pascal Leprince, Paul Achouch, Eloi Marijon, Alexandre Loupy, Xavier Jouven

**Affiliations:** Université de Paris, Paris Cardiovascular Research Center (PARCC), Paris Translational Research Center for Organ Transplantation, INSERM, UMR-S970, 75015 Paris, France; UMCV, Haut-Lévêque Hospital, University Hospital of Bordeaux, 33600 Pessac, France; Université de Paris, Paris Cardiovascular Research Center (PARCC), Paris Translational Research Center for Organ Transplantation, INSERM, UMR-S970, 75015 Paris, France; Department of Cardiac and Thoracic Surgery, Cardiology Institute, Pitié-Salpeêtrière Hospital, Assistance Publique des Hôpitaux de Paris (AP-HP), Sorbonne University Medical School, Paris, France; Université de Paris, Paris Cardiovascular Research Center (PARCC), Paris Translational Research Center for Organ Transplantation, INSERM, UMR-S970, 75015 Paris, France; Kidney Transplant Department, Necker Hospital, Assistance Publique—Hôpitaux de Paris, Paris, France; Université de Paris, Paris Cardiovascular Research Center (PARCC), Paris Translational Research Center for Organ Transplantation, INSERM, UMR-S970, 75015 Paris, France; Cardiology and Heart Transplant department, European Georges Pompidou Hospital, Assistance Publique—Hôpitaux de Paris, Rue Leblanc, 75015 Paris, France; Université de Paris, Paris Cardiovascular Research Center (PARCC), Paris Translational Research Center for Organ Transplantation, INSERM, UMR-S970, 75015 Paris, France; Université de Paris, Paris Cardiovascular Research Center (PARCC), Paris Translational Research Center for Organ Transplantation, INSERM, UMR-S970, 75015 Paris, France; Cardiology and Heart Transplant department, European Georges Pompidou Hospital, Assistance Publique—Hôpitaux de Paris, Rue Leblanc, 75015 Paris, France; Cardiology and Heart Transplant department, European Georges Pompidou Hospital, Assistance Publique—Hôpitaux de Paris, Rue Leblanc, 75015 Paris, France; Pathology Department, Georges Pompidou Hospital, Assistance Publique—Hôpitaux de Paris. Université de Paris, Paris, France; Department of Cardiac and Thoracic Surgery, Cardiology Institute, Pitié-Salpeêtrière Hospital, Assistance Publique des Hôpitaux de Paris (AP-HP), Sorbonne University Medical School, Paris, France; INSERM, UMRS-1166, iCAN, Institute of Cardiometabolism and Nutrition, Hôpital de la Pitié-Salpêtrière, Assistance Publique-Hôpitaux de Paris, Université Pierre et Marie Curie, Paris, France; Department of Cardiac and Thoracic Surgery, Cardiology Institute, Pitié-Salpeêtrière Hospital, Assistance Publique des Hôpitaux de Paris (AP-HP), Sorbonne University Medical School, Paris, France; INSERM, UMRS-1166, iCAN, Institute of Cardiometabolism and Nutrition, Hôpital de la Pitié-Salpêtrière, Assistance Publique-Hôpitaux de Paris, Université Pierre et Marie Curie, Paris, France; Cardiology and Heart Transplant department, European Georges Pompidou Hospital, Assistance Publique—Hôpitaux de Paris, Rue Leblanc, 75015 Paris, France; Université de Paris, Paris Cardiovascular Research Center (PARCC), Paris Translational Research Center for Organ Transplantation, INSERM, UMR-S970, 75015 Paris, France; Cardiology and Heart Transplant department, European Georges Pompidou Hospital, Assistance Publique—Hôpitaux de Paris, Rue Leblanc, 75015 Paris, France; Université de Paris, Paris Cardiovascular Research Center (PARCC), Paris Translational Research Center for Organ Transplantation, INSERM, UMR-S970, 75015 Paris, France; Kidney Transplant Department, Necker Hospital, Assistance Publique—Hôpitaux de Paris, Paris, France; Université de Paris, Paris Cardiovascular Research Center (PARCC), Paris Translational Research Center for Organ Transplantation, INSERM, UMR-S970, 75015 Paris, France; Cardiology and Heart Transplant department, European Georges Pompidou Hospital, Assistance Publique—Hôpitaux de Paris, Rue Leblanc, 75015 Paris, France

**Keywords:** Heart transplantation, Sudden cardiac death, Donor-specific antibodies

## Abstract

**Aims:**

The epidemiology of sudden cardiac death (SCD) after heart transplantation (HTx) remains imprecisely described. We aimed to assess the incidence and determinants of SCD in a large cohort of HTx recipients, compared with the general population.

**Methods and results:**

Consecutive HTx recipients (*n* = 1246, 2 centres) transplanted between 2004 and 2016 were included. We prospectively assessed clinical, biological, pathologic, and functional parameters. SCD was centrally adjudicated. We compared the SCD incidence beyond the first year post-transplant in this cohort with that observed in the general population of the same geographic area (registry carried out by the same group of investigators; *n* = 19 706 SCD). We performed a competing risk multivariate Cox model to identify variables associated with SCD. The annual incidence of SCD was 12.5 per 1,000 person-years [95% confidence interval (CI), 9.7–15.9] in the HTx recipients cohort compared with 0.54 per 1,000 person-years (95% CI, 0.53–0.55) in the general population (*P* < 0.001). The risk of SCD was markedly elevated among the youngest HTx recipients with standardized mortality ratios for SCD up to 837 for recipients ≤30 years. Beyond the first year, SCD was the leading cause of death. Five variables were independently associated with SCD: older donor age (*P* = 0.003), younger recipient age (*P* = 0.001) and ethnicity (*P* = 0.034), pre-existing donor-specific antibodies (*P* = 0.009), and last left ventricular ejection fraction (*P* = 0.048).

**Conclusion:**

HTx recipients, particularly the youngest, were at very high risk of SCD compared with the general population. The consideration of specific risk factors may help identify high-risk subgroups.

## Graphical Abstract

**Graphical abstract:**
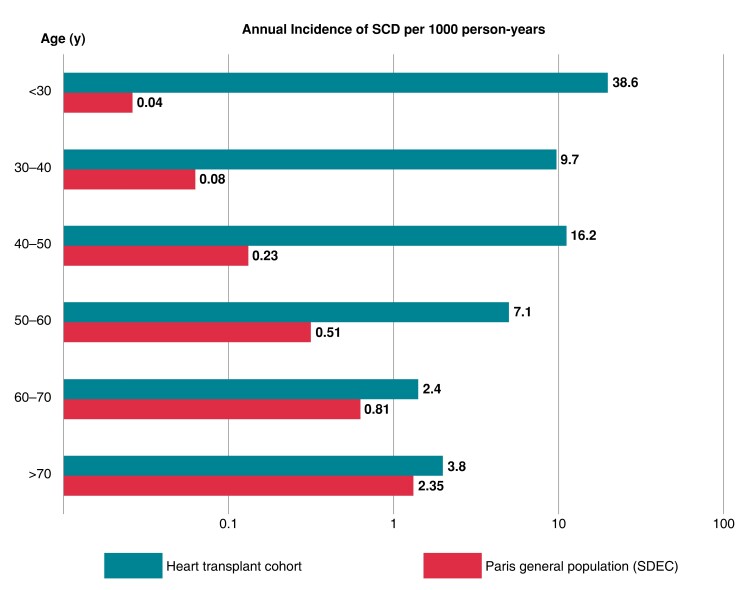

**What’s new?**

- Sudden cardiac death (SCD) represents a significant cause of late mortality after heart transplantation.
- Two large prospective registries from the same geographic area and carried out by the same group of investigators were analysed: (i) a cohort of 1246 consecutive heart transplant recipients including recent immunologic parameters and (ii) a cohort of 19 706 SCD from the prospective Paris Sudden Death Expertise Center registry.
- The annual incidence of SCD was 12.5 per 1000 person-years after heart transplantation compared to 0.54 in the general population. Beyond the first year post-transplant, SCD was the leading cause of death in this population. The risk of SCD was more elevated among the youngest HTx recipients.
- Five variables were independently associated with SCD: older donor age, younger recipient age and ethnicity, pre-existing donor-specific antibodies, and left ventricular ejection fraction.

## Introduction

The prevalence of heart failure continues to rise over time with the ageing of the population. Currently, 64.3 million individuals are affected by heart failure worldwide.^1^ Despite major advances in pharmacological and interventional treatments, heart transplantation (HTx) remains the ultimate treatment for terminal heart failure. Approximately 5500 HTx are performed annually worldwide,^2^ and post-transplant survival now exceeds 12 years. This progressive improvement in post-transplant outcome has mainly been driven by the decrease in early post-transplant mortality, while survival beyond the first year post-HTx has remained unchanged over time. The prevention of complications that can compromise long-term patient survival is an unmet medical need.

In this setting, sudden cardiac death (SCD) has been suggested to be an important cause of late mortality after HTx.^3^ Different underlying mechanisms of SCD are possible, but a primary arrhythmic origin is involved in most cases in the general population.^4^ However, several issues have severely hampered a thorough understanding of, including a limited numbers of patients, a short follow-up, poorly phenotyped cohorts, and large registries and administrative data lacking granularity and comparison with non-HTx patients.^5^ Together, these shortcomings have led to imprecise epidemiology and risk stratification of SCD after HTx.^6^ Therefore, an evaluation of the incidence of SCD after HTx and the identification of its specific risk factors including immunological factors based on large cohorts of heart recipients may improve patient monitoring and management.

In this study, we aimed to assess the incidence and determinants of SCD in a large cohort of HTx recipients, compared with the general population.

## Methods

Data, methods, and materials used to conduct the research are available from the corresponding author upon reasonable request.

### Heart transplant cohort

All consecutive HTx recipients over 18 years of age between 1 January 2004 and 31 December 2016 were enrolled in the multi-centre prospective Paris Transplant Group cohort (La Pitié-Salpêtrière Hospital, Paris, and Georges Pompidou European Hospital, Paris), which performed 72.8% of all HTx procedures performed during this period in Paris and its suburbs. We focused our analysis on SCD beyond the first year post-transplant as (i) the epidemiology of early deaths is very different from late deaths, both concerning the incidence (higher risk of death during the early post-transplant period) and the causes of death^2,6^ (early deaths mainly due to primary graft dysfunction and infection); (ii) early SCD are, in our experience, mostly in-hospital SCD, thus raising concerns about the comparison of the incidence of SCD with the general population; and (iii) to avoid survival bias when evaluating the effect of rejection and cardiac allograft vasculopathy (CAV) because patients who die early post-transplant may not have had the opportunity to have rejection or be diagnosed with CAV.

The design of this prospective cohort has been described previously.^7^ All data were anonymized and entered at the time of transplantation, at 3 months, 6 months, 1 year post-transplant, and at the subsequent annual visit evaluations using a standardized protocol to ensure harmonization across study centres. In addition, clinically driven events such as symptomatic rejection were collected. The day of transplant and 1 year post-transplant visits included an extensive clinical, biological, and functional evaluation (see Supplementary material online, *Appendix* for detailed data collection procedures). Data were retrieved from the database in December 2019. The study was conducted in accordance with the ethical standards laid down in the 1964 Declaration of Helsinki and its later amendments. The institutional review board of the Paris Transplant Group (https://paristxgroup.weebly.com/#) approved the study.

### Procedure and clinical protocols

All patients were followed from HTx until retransplantation, death, loss of follow-up, or date of final data extraction. Patient baseline information included donor characteristics, baseline recipient characteristics, immunological treatments, transplantation parameters, immunological parameters, histological data, interventional, and echocardiographic reports (see Supplementary material online, *Appendix*). Circulating donor-specific antibodies (DSAs) against human leucocyte antigen (HLA)-A, HLA-B, HLA-Cw, HLA-DR, HLA-DQ, and HLA-DP were assessed using single-antigen flow bead assays as described previously (see Supplementary material online, *Supplementary Methods*). Endomyocardial biopsies (EMBs) were performed, processed, and examined according to current standards. The EMB results were classified as cellular (Acute cellular rejection (ACR) 0–3R) or antibody-mediated rejection (AMR, pAMR 0–3) according to international guidelines (*n* = 13 676). The detailed routine EMB protocol and the histological analysis are detailed in the Supplementary material online, *Supplementary Methods*. Echocardiography was routinely performed on all patients by a senior cardiologist, and the left ventricular ejection fraction (LVEF) was reported and established by the biplane method of disk summation (modified Simpson’s rule). CAV angiographies were recorded per centre protocol for all patients after transplantation. The routine evaluation included an initial evaluation at 1-year post-transplant followed by additional evaluations every other year or in the case of clinical indication. CAV was graded according to international classification as CAV 0 (not significant), 1 (mild), 2 (moderate), or 3 (severe). The last left ventricular ejection fraction (LVEF) measure and the CAV grade were considered as potential risk factors for SCD.

### Adjudication of the causes of death

A pre-specified protocol was applied to analyse the causes of death by cross-checking multiple sources of data including medical reports, death certificates, and information reported by the general practitioner and the family (see Supplementary material online, *Supplementary Methods*). Each cause of death was centrally adjudicated by two senior cardiologists blinded to patient characteristics (G.B., G.C.). In the case of discrepancies between the observers or an inconclusive report, the cases were re-analysed to reach an agreement with a third expert. The causes of death were classified as follows: infection, graft failure (including primary graft dysfunction and late graft failure due to rejection, CAV or from unknown causes), SCD, cardiovascular (including major adverse cardiovascular events), malignancy, others (including renal failure, bleeding complications, multi-organ failure and surgical complications), and unknown causes. According to the latest international guidelines,^8^ we strictly defined SCD as an unexpected sudden death without obvious extracardiac cause, occurring with a rapid witnessed collapse within 1 h after the onset of symptoms, or if unwitnessed, occurring within 24 h after the last contact, in the absence of a prior terminal condition. The methodology of adjudication and the definition of sudden death were comparable between the HTx cohort and the Paris Sudden Death Expertise Center (Paris-SDEC) registry. One of the investigators (G.B.) who adjudicated the cause of death in the transplant cohort is also involved in the adjudication process at the Paris-SDEC.

### Paris Sudden Death Expertise Center registry

To compare the incidence of SCD, we used data from the Paris-SDEC registry, a prospective, population-based registry that recruits all consecutive SCD in the general population, in the same geographic area [Paris and its suburbs, comprising 6.7 million inhabitants, (approximately 10% of the total French population)],^9^ and is co-ordinated by the same group of investigators (Paris Cardiovascular Research Center). This registry is an ongoing study that has been described previously. Briefly, it is a comprehensive, prospective, population-based registry comprising Paris and its suburbs. Owing to a close collaboration with all the pre-hospital emergency medical services, 48 hospitals and forensic units, every case of out-of-hospital cardiac arrest aged ≥ 18 years occurring in the area has been enrolled systematically in the SDEC registry since May 2011. Exclusion criteria include age less than 18 years and cardiac arrest occurring outside the geographical area of interest. Regular external audits of the registry have shown that 99% of cardiac arrest cases are detected. The diagnosis of SCD was centrally adjudicated according to the definition described above by two independent investigators. In cases of a divergent opinion, a third expert was asked to arbitrate.

To calculate the annual incidence of SCD in the general population, we combined data from the Paris-SDEC with regional demographical data from the French National Institute of Statistics and Economic Studies. We included patients from May 2011 to May 2017.

### Statistical analysis

We performed the statistical analysis in compliance with the Strengthening the Reporting of Observational Studies in Epidemiology checklist for observational studies.^10^ Continuous variables are described as the mean and standard deviation (SD) for normally distributed data and as the median and interquartile range (IQR) for non-normally distributed data. Categorical variables are reported using absolute and relative frequency distributions. We compared means and proportions between groups by using Student’s *t*-test, analysis of variance (Mann–Whitney test for mean fluorescence intensity), or the χ^2^ test (or Fisher’s exact test if appropriate).

### Incidence of sudden cardiac death

The incidence of SCD per 1000 person-years was calculated for the entire cohort and then after stratification by age categories for both the HTx cohort and the general population. The incidence of SCD between cohorts was compared using the Mid-P Fisher’s exact test. The standardized mortality ratio was computed by dividing the observed number of SCD in the cohort of HTx patients by the expected number based on rates observed in the general population (Paris-SDEC registry).

### Competing risk models

Causes of death and respective cumulative incidences were analysed by using competing risk models. The cumulative incidence function represents the probability of occurrence by time of a particular type of death in the presence of other causes of death. The survival analysis was performed from 1 year after HTx to the maximum follow-up with SCD as the event of interest. Survival probability was assessed with Kaplan–Meier curves and compared using a log-rank test. Censoring occurred in the event of loss to follow-up, heart re-transplantation, or non-sudden death.

### Determinants of sudden cardiac death

Cox proportional hazards models were applied to identify the clinical, immunological, histological, echocardiographic and angiographic parameters independently associated with SCD. In the univariate analysis, parameters were selected on the result of bivariate analyses (variables with a *P* value <0.1) and clinical relevance. The final multivariate model was selected based on the stepwise backward selection, with the competitive risk modelling of Fine and Gray. A two-tailed *P* < 0.05 was considered statistically significant. All data were analysed using STATA (version 15, Data Analysis and Statistical Software) and R software (version 3.6.3, R Project for Statistical Computing, Vienna, Austria).

## Results

### Study population

A total of 1220 patients were included from two French referral HTx centres (*n* = 1024 in La Pitié-Salpêtrière Hospital, Paris, and *n* = 222 in Georges Pompidou European Hospital, Paris, Supplementary material online, *Table S1*). During the first-year post-transplant, 315 patients died, mostly due to infection (*n* = 112, 35.6% of causes of death) and graft failure (*n* = 82, 26.0% of causes of death, Supplementary material online, *Figure S1*), leaving 905 patients alive at 1 year post-transplant. Their baseline characteristics are presented in *Table 1*. The mean recipient age at transplant was 47.9 ± 13 years, and the mean donor age was 45.4 ± 13.2 years. Two thirds of the recipients were males. A total of 261 patients (30%) were transplanted with pre-existing DSA. The median post-transplant follow-up was 6.8 years (IQR 4.2–10.1). A total of 209 (23.1%) patients died during follow-up. The overall 5- and 10-year survival rates for those who had survived after the first year were 83.6% [95% confidence interval (CI): 80.8–86.1] and 67.7%, (95% CI: 63.3–71.8) respectively. During follow-up, 35 patients (3.9%) were implanted with a pacemaker or defibrillator. Among them, the recordings showed 48.5% (*n* = 17/34) paroxysmal atrial fibrillation (AF) and 8.6% (*n* = 3/35) non-sustained ventricular tachycardia. Among patients with an ICD (*n* = 6), one patient received an appropriate shock in the context of an electrical storm; no inappropriate ICD shock was recorded.

**Table 1:** Characteristics of patients among the 1-year survivors after transplantation

	1 year survivors	*N*
	*N* = *905*	
Donor characteristics		
Age (years), mean (SD)	45.4 (13.2)	905
Gender (men), *n* (%)	597 (66.0)	905
Tobacco, *n* (%)	315 (45.8)	688
Hypertension, *n* (%)	123 (17.9)	689
Diabetes mellitus, *n* (%)	19 (3.13)	607
Alcohol, *n* (%)	214 (31.1)	689
BMI (kg/m^2^), mean (SD)	25.4 (4.79)	902
Creatinine clearance (mL/min/1.73 m^2^), mean (SD)	99.5 (25.4)	605
Recipient characteristics		
Age (years), mean (SD)	47.9 (13.0)	905
Gender (men), *n* (%)	711 (78.6)	905
BMI (kg/m^2^), mean (SD)	24.3 (4.16)	905
Non-Caucasian ethnicity, *n* (%)	255 (28.2)	905
Primary heart disease, *n* (%)		905
Dilated cardiomyopathy	404 (44.6)	
Ischaemic cardiomyopathy	285 (31.5)	
Re-transplantation	21 (2.32)	
Congenital	44 (4.86)	
Other	151 (16.7)	
Hypertension, *n* (%)	282 (32.2)	875
Diabetes mellitus, *n* (%)	144 (16.0)	902
History of smoking, *n* (%)	475 (53.1)	895
Long-term MCS, *n* (%)	122 (13.5)	905
ECMO at transplant, *n* (%)	216 (23.9)	905
Transplant baseline characteristics		
Cold ischaemic time (min), mean (SD)	185 (58.2)	905
Induction therapy, *n* (%)		905
ATG	834 (92.2)	
Others	10 (1.1)	
IL2-R inhibitor	61 (6.7)	
Gender mismatch (Df Rm), *n* (%)	192 (21.2)	905
Combined transplantation, *n* (%)	43 (4.8)	905
CMV mismatch (D + R−), *n* (%)	171 (19.4)	883
Immunology and histology		
HLA mismatches (A/B/DR), mean (SD)	4.99 (0.96)	880
Pre-formed DSA, *n* (%)	261 (29.0)	899
ACR ≥ 1B at 1 year, *n* (%)	424 (47.2)	898
ACR ≥ 2R at 1 year, *n* (%)	124 (13.8)	
AMR at 1 year, *n* (%)	75 (8.34)	899

ACR, acute cellular rejection; AMR, antibody-mediated rejection; ATG, anti-thymocyte globulin; BMI, body mass index; CI, confidence interval, presented as [lower limit; upper limit]; CMV, cytomegalovirus; Df Rm, femal donor and male recipient; DSA, donor-specific antibodies; ECMO, extracorporeal membrane oxygenation; HTx, heart transplantation; ID, immunodominant; IL2-r, interleukin-2 receptor; LVEF, left ventricular ejection fraction; MCS, mechanical circulatory support; MFI, mean fluorescence intensity; MMF, mycophenolate mofetil.

### Causes of death after the first year post-transplant and the incidence of sudden cardiac death

**Figure 1:**
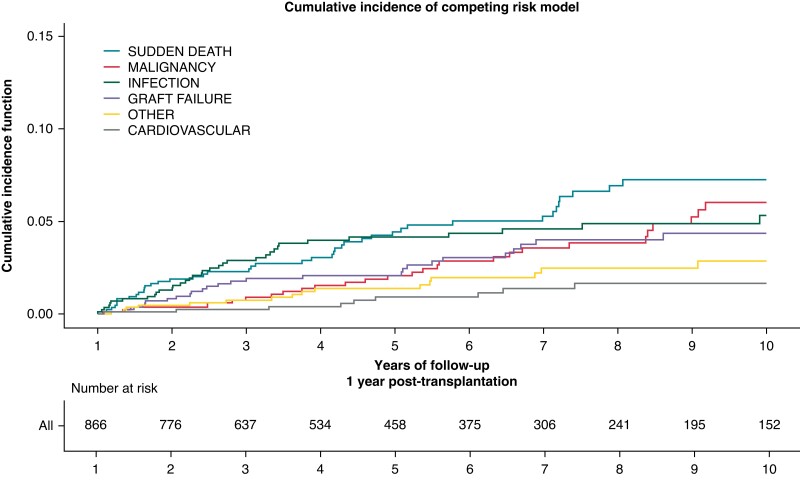
Cumulative incidence of death causes in heart transplant recipients beyond the first year (*n* = 905). *The curves represent the cumulative incidence of death by cause in patients who survived beyond the first year: sudden cardiac death, malignancy, infection, graft failure (including primary graft dysfunction and late graft failure due to rejection, cardiac allograft vasculopathy, or from others causes), cardiovascular (including major adverse cardiovascular events), and other (including renal failure, bleeding complications, multi-organ failure, and surgical complications).

**Figure 2:**
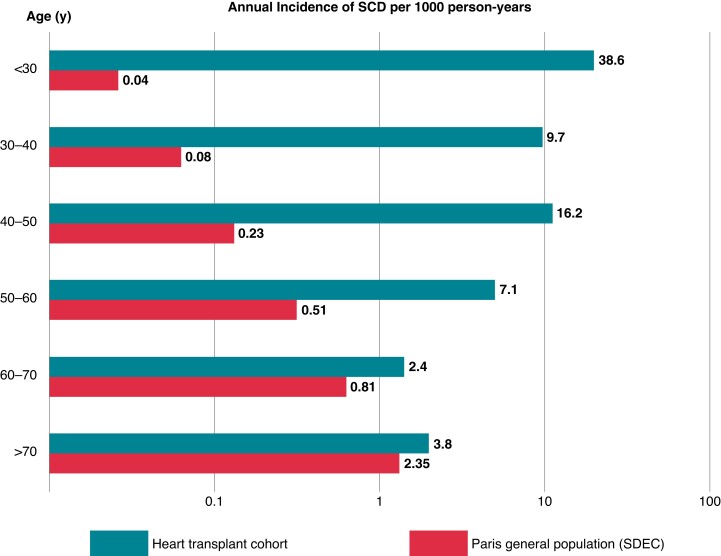
Annual incidence of sudden cardiac death according to patient’s age in the Paris general population [sudden death expertise centre (SDEC) registry] and in the heart transplant cohort. The annual incidence of sudden death was represented by 10-year age groups in the Paris general population through the Paris-SDEC and in the heart transplant patients in the same geographical area over the same period. The incidences have been logarithmically transformed.

The cumulative incidence of the different causes of death after the first year post-transplant is presented in *Figure 1*. Overall, 46 deaths met the definition criteria of SCD, representing 22% of causes of death. SCD was the leading cause of death, ahead of malignancy and infection. The median time between HTx and SCD was 5.3 years (IQR 2.9–8.6). The characteristics of patients with SCD and non-sudden death are provided in Supplementary material online, *Table S2*. The crude annual incidence of SCD was 12.5 per 1000 person-years (95% CI 9.7–15.9). We observed a stepwise increase in the risk of SCD with decreasing recipient age (incidence of SCD = 38.6 per 1000 person-years in recipients <30 years at transplant compared with 3.8 per 1000 person-years in those >70 years at transplant, *Figure 2*).

### Comparison of the incidence of sudden cardiac death with the general population

In the Paris-SDEC registry, there were a total of 23 086 out-of-hospital cardiac arrests from May 2011 to May 2017, among which 19 706 cases fit the SCD definition. The mean age of patients presenting SCD in the general population was 70.8 ± 16.9 years, and 14 105 (61.1%) were male. Considering the average number of inhabitants in this area during the same period, the crude annual incidence of SCD was 0.54 per 1000 person-years (95% CI 0.53–0.55). The incidence of SCD was significantly higher for HTx recipients compared with the general population from the same geographic area (*P* < 0.001).

In contrast to the HTx recipients cohort, the incidence of SCD was higher in older patients in the general population (0.04 per 1000 person-years in patients <30 years compared with 2.4 per 1000 person-years in those >70 years, *Figure 2*). The corresponding standardized mortality ratio increased from 1.3 in HTx patients >70 years to 2.2 between 60 and 70 years, 10.8 between 50 and 60 years, 53.7 between 40 and 50 years, 107.9 between 30 and 40 years, and 837.6 in patients <30 years.

### Identification of the determinants of sudden cardiac death after heart transplantation

Variables associated with SCD beyond the first year post-HTx after the univariate Cox analysis are reported in *Table 2* and included donor age [per 10-year increment, hazard ratio (HR) 1.34, 95% CI: 1.04–1.74], recipient age (per 10-year increment, HR 0.73, 95% CI 0.60–0.90), non-Caucasian ethnicity of the recipient (HR 2.35, 95% CI 1.30–4.25), presence of pre-existing anti-HLA DSA (HR 2.78, 95% CI 1.52–5.09), AMR pAMR2 (HR 9.97, 95% CI 3.08–32.3), last LVEF evaluation (per 10% decrement, HR 1.43, 95% CI 1.05–1.92), and the CAV grade at 4 years post-transplant (*P* = 0.009). Thirty-five percent of patients with impaired left ventricular function (LVEF < 45%) had severe CAV (grade 2–3 CAV).

**Table 2:** Factors associated with sudden cardiac death in the univariate Cox analysis in 1-year survivors

	All patients	No SCD	SCD	HR (95% CI)	*P* value	*n*
	*n* = 905	*n* = 859	*n* = 46			
**Donor characteristics**						
Age (years), mean (SD)	45.4 (13.2)	45.1 (13.3)	49.3 (10.8)	1.34 (1.04; 1.74)^a^	0.026	905
Gender (men), *n* (%)	597 (66.0)	564 (65.7)	33 (71.7)	1.32 (0.69; 2.51)	0.397	905
Tobacco, *n* (%)	315 (45.8)	301 (45.8)	14 (45.2)	1.02 (0.50; 2.07)	0.961	688
Hypertension, *n* (%)	123 (17.9)	119 (18.1)	4 (12.9)	0.67 (0.23; 1.91)	0.448	689
Diabetes mellitus, *n* (%)	19 (3.1)	19 (3.3)	0 (0.0)	—	0.495	607
Alcohol, *n* (%)	214 (31.1)	202 (30.7)	12 (38.7)	1.25 (0.60; 2.57)	0.548	689
BMI (kg/m^2^), mean (SD)	25.4 (4.8)	25.4 (4.8)	24.6 (4.1)	0.97 (0.91; 1.03)	0.313	902
eGFR (mL/min/1.73 m^2^), mean (SD)	99.4 (25.4)	99.2 (25.6)	107 (19.2)	1.02 (1.00; 1.04)	0.096	605
Chronic kidney failure, *n* (%)	55 (9.1)	55 (9.4)	0 (0.0)	—	0.138	605
**Recipient characteristics**						
Age (years), mean (SD)	47.9 (13.0)	48.3 (12.8)	40.7 (14.2)	0.73 (0.60; 0.90)^a^	0.003	905
Gender (men), *n* (%)	711 (78.6)	678 (78.9)	33 (71.7)	0.74 (0.39; 1.40)	0.352	905
BMI (kg/m^2^), mean (SD)	24.3 (4.2)	24.3 (4.1)	24.8 (4.8)	1.04 (0.97; 1.11)	0.301	905
Non-Caucasian ethnicity, *n* (%)	255 (28.2)	236 (27.5)	19 (41.3)	2.35 (1.30; 4.25)	0.004	905
Primary heart disease, *n* (%)						
Dilated cardiomyopathy	404 (44.6)	388 (45.2)	16 (34.8)	Reference	0.089	905
Ischaemic cardiomyopathy	285 (31.5)	274 (31.9)	11 (23.9)	1.03 (0.48; 2.23)		
Re-transplantation	21 (2.32)	20 (2.33)	1 (2.17)	1.57 (0.21; 11.8)		
Congenital	44 (4.86)	39 (4.54)	5 (10.9)	2.78 (1.02; 7.62)		
Other	151 (16.7)	138 (16.1)	13 (28.3)	2.16 (1.04; 4.50)		
Hypertension, *n* (%)	282 (32.2)	267 (32.1)	15 (34.9)	1.14 (0.60; 2.14)	0.691	875
Diabetes mellitus, *n* (%)	144 (16.0)	136 (15.9)	8 (17.4)	1.15 (0.54; 2.48)	0.714	902
History of smoking, *n* (%)	475 (53.1)	455 (53.6)	20 (43.5)	0.84 (0.47; 1.51)	0.567	895
Long-term MCS, *n* (%)	122 (13.5)	122 (14.2)	0 (0.0)	—	0.010	905
ECMO at transplant, *n* (%)	216 (23.9)	204 (23.7)	12 (26.1)	1.32 (0.68; 2.55)	0.416	905
**Transplant baseline characteristics**						
Cold ischaemic time (min), mean (SD)	185 (58.2)	184 (58.4)	192 (53.8)	1.00 (1.00; 1.01)	0.242	905
Induction therapy, *n* (%)					0.841	905
ATG	834 (92.2)	792 (92.2)	42 (91.3)	Reference		
IL2-r inhibitor	61 (6.7)	58 (6.8)	3 (6.5)	1.06 (0.33; 3.42)		
Other	10 (1.1)	9 (1.1)	1 (2.2)	1.79 (0.25; 13.1)		
Gender mismatch (Df Rm), *n* (%)	192 (21.2)	184 (21.4)	8 (17.4)	0.79 (0.37; 1.70)	0.554	905
Combined transplantation, *n* (%)	43 (4.8)	40 (4.7)	3 (6.52)	1.70 (0.53; 5.51)	0.371	905
CMV mismatch (D + R−), *n* (%)	171 (19.4)	166 (19.8)	5 (11.4)	0.47 (0.18; 1.20)	0.106	883
**Immunology and histology**						
HLA mismatches (A/B/DR), mean (SD)	4.99 (1.0)	4.98 (1.0)	5.20 (0.9)	1.28 (0.91; 1.79)	0.157	880
Pre-formed DSA, *n* (%)	261 (29.0)	242 (28.4)	19 (41.3)	2.78 (1.52; 5.09)	0.001	899
**Histology during the first year post-transplant**						
TCMR ≥ 1R1B, *n* (%)	424 (47.2)	400 (46.9)	24 (53.3)	1.22 (0.67; 2.21)	0.507	898
Number of TCMR ≥ 1R1B, mean (SD)	0.93 (1.3)	0.92 (1.3)	1.13 (1.4)	1.08 (0.89; 1.32)	0.434	898
TCMR ≥ 2R, *n* (%)	124 (13.8)	115 (13.5)	9 (20.0)	1.43 (0.68; 2.97)	0.343	898
TCMR ≥ 3R, *n* (%)	20 (2.2)	19 (2.2)	1 (2.2)	0.65 (0.09; 4.76)	0.675	898
AMR, *n* (%)	75 (8.3)	69 (8.1)	6 (13.3)	2.04 (0.86; 4.84)	0.098	899
Number of AMR, mean (SD)	0.14 (0.6)	0.13 (0.6)	0.40 (1.3)	1.46 (1.17; 1.82)	0.001	899
pAMR grade ≥ 2, *n* (%)	9 (1.0)	6 (0.7)	3 (6.7)	9.97 (3.08; 32.30)	<0.001	899
**Echocardiography parameters**						
Last LVEF (%), mean (SD)	64.9 (8.30)	65.0 (8.24)	62.2 (8.99)	1.43 (1.05; 1.92)^b^	0.020	885
**CAV distribution**						
CAV at 1 year, *n* (%)					0.196	769
Grade 0	628 (81.7)	597 (81.8)	31 (79.5)	Reference		
Grade 1	110 (14.3)	104 (14.2)	6 (15.4)	1.39 (0.58; 3.36)		
Grade 2	27 (3.5)	26 (3.6)	1 (2.6)	0.69 (0.09; 5.04)		
Grade 3	4 (0.52)	3 (0.41)	1 (2.56)	6.12 (0.83; 45.00)		
CAV at 4 years, *n* (%)					0.009	509
Grade 0	283 (55.6)	277 (56.5)	6 (31.6)	Reference		
Grade 1	155 (30.5)	145 (29.6)	10 (52.6)	5.15 (1.75; 15.1)		
Grade 2	55 (10.8)	53 (10.8)	2 (10.5)	3.10 (0.60; 16.0)		
Grade 3	16 (3.1)	15 (3.1)	1 (5.2)	6.08 (0.71; 52.2)		

AMR, antibody-mediated rejection; ATG, antithymocyte globulin; BMI, body mass index; CAV, cardiac allograft vasculopathy; CI, confidence interval, presented as (lower limit; upper limit); CMV, cytomegalovirus; DSA, donor-specific antibodies; ECMO, extracorporeal membrane oxygenation; eGFR, estimated Glomerular filtration rate; HTx, heart transplantation; ID, immunodominant; IL2-r, interleukin-2 receptor; LVEF, left ventricular ejection fraction; MCS, mechanical circulatory support; MFI, mean fluorescence intensity; MMF, mycophenolate mofetil; TCMR, T-cell–mediated rejection.

a 10-year increment.

b 10% decrement.

In the competing risk multivariate analysis, five variables were independently associated with SCD (*Table 3*): donor age (per 10-year increment, HR 1.44, 95%CI 1.13–1.83, *P* = 0.003), recipient age (per 10-year increment, HR 0.71, 95%CI 0.57–0.87, *P* = 0.001), non-Caucasian ethnicity of the recipient (HR 1.88, 95%CI 1.05–3.38, *P* = 0.034), presence of pre-existing anti-HLA DSA (HR 2.28, 95%CI 1.22–4.26, *P* = 0.009), and last LVEF evaluation (per 10% decrement, HR 1.32, 95%CI 1.01–1.69, *P* = 0.048). The incidence of sudden death for each sub-population group defined by the presence of one of the risk factors is reported in Supplementary material online, *Table S3*.

**Table 3:** Factors associated with sudden cardiac death in the multivariable Cox analysis with the competitive risk model

*N* = 885	HR (95% CI)	*P* value
**Donor characteristics**		
Age (per 10-year increment)	1.44 (1.13; 1.83)	0.003
**Recipient characteristics**		
Age (per 10-year increment)	0.71 (0.57; 0.87)	0.001
Non-Caucasian ethnicity	1.88 (1.05; 3.38)	0.034
**Immunology**		
Pre-formed DSA	2.28 (1.22; 4.26)	0.009
**Echocardiography parameters**		
Last LVEF (per 10% decrement)	1.32 (1.01; 1.69)	0.048

This table shows the clinical, immunological, functional and structural parameters associated with sudden cardiac death in the multivariable Cox analysis, with competitive risks taking into account all other known causes of death.

DSA, donor-specific antibodies; LVEF, left ventricular ejection fraction.

### Sensitivity analyses

Various sensitivity analyses were performed to test the robustness of the final model of candidate variables associated with SCD.

- *AMR grade >2 and sudden death:* When we forced histological AMR grade ≥ 2 in the final multivariable model instead of pre-formed DSA, it showed an association (*P* = 0.001) with SCD (see Supplementary material online, *Table S4*), but it was outperformed by the circulating anti-HLA DSA status.
- *Multivariate analysis with competitive risks of all cause of deaths including unknown causes of death:* By considering unknown causes of death as potential competitive risks, the final multivariate model remained unchanged (see Supplementary material online, *Table S5*).
- *Multivariate analysis stratified by centre:* To take into account a possible bias in the assessment of the determinants of the SCD, the centre was also entered in the final multivariable model and did not modify the set of independent parameters associated with SCD (see Supplementary material online, *Table S6*).

## Discussion

Based on the analysis of two large prospective cohorts, we showed that the risk of SCD was increased substantially in HTx recipients compared with the general population from the same geographic area. The youngest HTx recipients were at particularly high risk of SCD. In the competing risk analysis, SCD appeared to be the leading cause of death after the first year post-HTx. We identified five independent variables associated with SCD, including older age of the donor, younger age of the recipient, circulating DSA, LVEF, and ethnicity. Our study represents the first step towards an improvement in the description of the epidemiology and risk factors of SCD after HTx and has several strengths including the large prospective and deeply phenotyped cohort of HTx recipients, the large exhaustive registry of SCD in the general population from the same geographic area and a competing risk analysis that has allowed precise investigation of the specific determinants of SCD.

### High incidence of sudden cardiac death

In our cohort, SCD was the leading cause of death beyond the first year after HTx, accounting for 22% of all causes of death. Data from the UNOS registry suggest that SCD may be less prevalent in the USA.^11^ However, in a recent meta-analysis including 47 901 patients, the pooled incidence rate of SCD has been described to be as high as 13.0 per 1000 person-years (95% CI: 10.8–15.2) compared with 12.5 per 1000 person-years (95% CI: 9.7–15.9) post-transplant in our study,^6^ suggesting that we have not overestimated the incidence of SCD after HTx. Additionally, the lack of granularity of large international registries may lead to an underestimation of the exact incidence of SCD after HTx. Finally, the distribution of risk factors differed between our cohort and international data. Compared with the ISHLT registry, donors in our cohort were older, recipients were younger, and the proportion of patients transplanted with pre-formed DSA was as high as 30%.

Sudden cardiac death before and after heart transplantationIn patients with non-ischaemic cardiomyopathy and severe left ventricular dysfunction, the incidence of SCD was 15.0 per 1000 person-years and significantly reduced by an implantable cardiac defibrillator (ICD) in the DANISH trial. This observed threshold appears to be relatively consistent with other studies showing the benefit of ICD in preventing sudden death, both in the setting of ischaemic and non-ischaemic heart disease.^12,13^ In our cohort, all patients with at least one identified risk factor had an annual incidence of sudden death between 14.9 and 24.0 per 1000 person-years (see Supplementary material online, *Table S3*).

The mechanisms of SCD after HTx may significantly differ compared with pre-HTx SCD. Non-shockable rhythms (asystole or pulseless electrical activity) have been reported as important mechanisms of death in patients after HTx. However, the results of this study must be interpreted in the context of (i) the natural evolution from a shockable to a non-shockable rhythm, which may underestimate the prevalence of initial ventricular arrhythmias, and (ii) the unusually high proportion of in-hospital sudden death in this study, making these results likely not representative of all transplant recipients.

The identification of predictive variables independently associated with the risk of sudden death is a first step towards the identification of high-risk patients. While invasive preventive strategies based on ICD implantation are not recommended at the population level, our findings suggest that it may be interesting to continue the pre-HTx strategies after HTx to prevent SCD and that the identification of transplant-specific risk factors may help clinicians to screen for high-risk patients in whom aggressive preventive strategies should be discussed. The prevention of SCD after HTx is however particularly challenging and the potential impact of ICD in this setting remains to be evaluated, as electrocardiographic recordings at the time of SCD have shown a significant proportion of brady-arrhythmias, asystole, and electrical-mechanical dissociation.^6^

### Importance of patient age on the risk of sudden cardiac death

Compared with the general population, HTx recipients were at high risk of SCD. We found an opposite association between patient age and the risk of SCD in HTx recipients (a stepwise increase in the risk of SCD with decreasing recipient age) compared with the general population (a stepwise increase in the risk of SCD with increasing age). The increasing risk of SCD with patient age in the general population and with donor age in the HTx cohort is likely to reflect the increasing incidence of coronary artery disease with increasing age. On the other hand, the increased risk of SCD in young recipients may reflect the importance of underlying immune processes as a cause of allograft injury and arrhythmogenic substrate.

### Immune determinants of sudden cardiac death after heart transplantation

We found marginal associations between allograft rejection and the risk of SCD in our cohort, contrasting with the robust association with the presence of HLA-DSAs, which may be a more sensitive marker of allosensitization than AMR. Although acute and severe rejection has been associated with SCD, this entity is rare and chronic allograft inflammation induced by allosensitization may be a trigger of chronic allograft injury at the population level. Circulating HLA-DSAs have been shown to be a major determinant of premature and accelerated allograft fibrosis following kidney,^14^ liver,^15^ and HTx.^16^ Interestingly, myocardial fibrosis is a common pattern of various heart diseases that promotes ventricular tachyarrhythmias by creating a vulnerable substrate for re-entrant activity and by favouring the emergence of triggers.^17^ In HTx recipients, myocardial fibrosis has been shown to be an intermediate pro-arrhythmogenic substrate with various aetiologies,^18^ including CAV, allograft rejection, ischaemic time^19^ or donor-transmitted fibrosis.^20^

### Cardiac allograft vasculopathy and sudden cardiac death

Consistent with previously published data,^6^ we found a significant association between CAV and sudden death in univariate analysis. However, CAV was not independently associated with SCD in our study, in contrary to circulating anti-HLA DSA and LVEF, two variables that had usually not been included in previous epidemiological study of post-transplant SCD. DSA have been shown to be an independent risk factor of CAV^7^ and may be a more sensitive predictive variable at the population level. Among patients diagnosed with CAV, the presence of significant systolic graft dysfunction may reflect the severity of underlying coronary lesions and ischaemic myocardial damage and, therefore, be a stronger predictor of SCD than the presence of coronary stenosis alone. Additionally, CAV may have been underestimated as a component of sudden death due to the long interval between two coronary angiograms (thus underestimating the potential evolution of CAV between two tests) and the limited diagnostic value of standard angiograms without endocoronary imaging (under-diagnosis of early forms of CAV).

### Perspective in SCD prediction after heart transplantation

Risk stratification of SCD after HTx is an unmet medical need that should be strongly addressed.^21^ Different strategies may improve this risk stratification. First, large multi-centre registries of highly-phenotyped HTX recipients including transplant- (histology, immunology, biological) and non-transplant-specific variables are needed to build clinically-relevant risk models.^22^ Second, new tools such as machine learning may improve statistical performance of models since they have been shown to outperform traditional models in various areas.^23^ Third, the integration of signal analysis by artificial intelligence may represent a major advance in this our field.^24^ Altogether, these approaches might lead to better risk stratification and therefore earlier detection of high-risk patients.

### Limitations

Our study should be interpreted in the context of its limitations. First, despite a pre-specified protocol and exhaustive research, the precise cause of 49 out-of-hospital deaths (9.4%) could not be identified, including potential sudden deaths. Hence, the incidence of SCD may have been underestimated. However, this rate appeared to be low compared with previous studies. Second, the limited number of events did not allow us to develop an accurate predictive score. Nevertheless, in our cohort, the incidence of SCD in patients with at least one risk factor was greater than the commonly accepted thresholds for primary preventive ICD implantation in various cardiac conditions. This simple approach may be used in clinical practice to refine risk stratification of SCD. Third, we were not able to determine the precise mechanisms of SCD in our cohort because only a minority of patients had a pacemaker/ICD or underwent autopsy. Fourth, 1-year post-transplant mortality was higher in our cohort compared with North America standards. However, (i) the mortality rate was comparable to European HTx results,^25^ (ii) this also reflected high-risk transplantation following the 2004 update of the French allocation scheme (high emergency status),^26^ and (iii) we analysed causes of death beyond the first year post-transplant, making it unlikely that this high early mortality rate had a significant impact on our results. Finally, the absence of an independent association between biopsy-proven rejection or CAV and sudden death may be due to insufficiently sensitive tests to detect subclinical and chronic rejection.

## Conclusion

We found that the risk of SCD was substantially increased in HTx recipients compared with the general population. The youngest HTx recipients were at particularly high risk. Five independent variables were independently associated with the risk SCD.

## Supplementary Material

euad126_Supplementary_Data

## Data Availability

The data underlying this article will be shared on reasonable request to the corresponding author.
