# Supplementary material for: Sudden cardiac death after heart transplantation: a population-based study
Source: Europace. 2023 May 19;25(5):euad126. doi: 10.1093/europace/euad126 (PMC10198773; doi:10.1093/europace/euad126)
Supplement: euad126_Supplementary_Data [file euad126_supplementary_data.docx]

**Supplementary Appendix**

# **S1. Data collection procedures**

Baseline clinical data on the donors and recipients from Paris were obtained from the national registry CRISTAL (Agence de la Biomédecine, French National Agency for Organ Procurement). Codes were used to ensure strict donor and recipient anonymity and blinded assays. Informed consent was obtained from the participants at the time of transplantation. Anonymised data from these registries are prospectively entered by dedicated staff at specific time points for each patient (day of listing, day of transplant and at hospital discharge) and are updated annually thereafter. Longitudinal data from the day of transplantation to 10 years post-transplantation were retrieved based on a complete review of medical charts and the collection of clinical events. This retrospective data collection was performed by cardiologists at each centre and research assistants. We performed an extensive evaluation of potential risk factors that might be associated with SCD. We recorded the following data: i) donor cardiovascular profile, including age at donation, sex, cause of death (including vascular, traumatic, anoxia or other), donor comorbidities including hypertension, diabetes mellitus, dyslipidaemia, tobacco and alcohol consumption, body mass index, renal function, medical treatment, the presence of a cardiac arrest during initial management, cytomegalovirus (CMV) serology and coronary angiograms; ii) recipient pre-transplant cardiovascular profile and general characteristics including age at transplantation, sex, ethnicity, hypertension, diabetes mellitus, dyslipidaemia, tobacco and alcohol consumption, body mass index, use of mechanical cardiac support or implanted devices, the presence of extracorporeal membrane oxygenation support at the time of transplantation and primary heart disease; iii) transplantation characteristics including cold ischaemic time, type of transplantation (isolated heart transplantation [HTx] vs combined transplantation), sex mismatch, weight mismatch and CMV mismatch; iv) immunosuppressive therapies including the type of induction therapy and 1 year immunosuppressive regimen (type of calcineurin inhibitors, mycophenolate mofetil, everolimus, azathioprine, corticosteroids); v) immunological parameters including the number of HLA A-B-DR mismatches (and A-B-DR-DQ when available) and the presence of pre-formed donor-specific antibodies (DSA) at the time of transplantation; vi) histological parameters of endomyocardial biopsies (EMB) retrospectively reclassified according to the International Society for Heart and Lung Transplantation (ISHLT) 2004 and 2013 guidelines for cellular rejection and antibody-mediated rejection (AMR)^1,2^; and vii) echocardiographic parameters including the ventricular ejection fraction established by the biplane method of disks summation (modified Simpson’s rule), which is in line with the latest recommendations.^3^

In both centres, trans-thoracic echocardiography was performed at the time of each outpatient visit:

- Year 1: 15 times;
- Years 2 and 3: every other month;
- Years 4 and 5: every 4 months;
- > Year 5: every 6 months.

The CRISTAL database networks have been approved by the National French Commission for Bioinformatics Data and Patient Liberty: CRISTAL: CNIL, registration number: 363505, validated 3 April 1996.

# **S2. Circulating donor-specific antibodies**

# All patients included in the study were screened for the presence of circulating anti-HLA antibodies in sera collected before and at the time of HTx. Banked sera were reassessed retrospectively for anti-HLA antibodies between November 2017 and March 2018, using Luminex technology, at the reference histocompatibility laboratory in Paris (Saint-Louis Hospital). An expert immunologist (JLT) interpreted the results and determined whether each tested serum sample contained anti-HLA antibodies directed against the donor (DSA). Antibodies against HLA-A, HLA-B, HLA-Cw, HLA-DR, HLA-DQ and HLA-DP epitopes were tested using single-antigen flow bead assays (One Lambda, Inc., Canoga Park, CA, USA) on a Luminex platform, as described previously.^4^ All beads with a normalised mean fluorescence intensity (MFI) > 500 were considered positive. In patients with multiple anti-HLA DSA, the immunodominant anti-HLA DSA (the one with the highest MFI) was selected for analysis. HLA typing of the recipients was performed via molecular biology (Innolipa HLA typing kit, Innogenetics, Ghent, Belgium). For all HTx donors, tissue typing was initially performed using the microlymphocytotoxicity technique with One Lambda Inc. tissue typing trays at transplantation, with the results confirmed by molecular biology.

#

# **S3. Pathology and immunosuppression**

# Endomyocardial biopsies (EMB) were performed, processed and examined according to current standards.^5^ The routine EMB protocol included 13 protocol EMB during the first year (every 10 days from day 15 to month 2 [M2], every month from M2 to M6 and every month and a half from M6 to M12). Additional biopsies were performed if clinically indicated. Serial sections were cut from formalin-fixed paraffin-embedded EMB specimens and stained with haematoxylin, eosin and saffron for diagnosis. Immunofluorescence for C4d was performed (frozen sections; C4d monoclonal [Quidel Corporation, San Diego, CA 92121, USA] diluted 1/100; polyclonal rabbit anti-mouse conjugated to FITC, Dako). Only capillary staining for C4d was assessed. The EMB were classified as cellular (0 to 3R) or AMR (pAMR 0 to 3) according to the ISHLT guidelines.^6^ For the purpose of this study, a systematic retrospective analysis of all abnormal EMB (i.e. ACR ≥ 1R1B and/or pAMR ≥ 1), for-cause EMB and pre-specified protocol EMB (first, M1, M3, M6 and M12) was performed by expert pathologists, independently of the initial pathological diagnosis and clinical data.

The immunosuppressive protocol has been reported previously.^7-9^

# **S4. Definition and criterion of causes of death**

Causes of death were defined in accordance with the causes commonly established in the ISHLT reports^10^: major adverse cardiovascular events (MACE) such as stroke, haemorrhage, cardiac allograft vasculopathy, malignancy, primary graft dysfunction, late graft dysfunction, rejection, infection (non-CMV), renal failure, multi-organ failure, mechanical or surgical complications, sudden death, other and unknown causes.

A pre-specified protocol was applied to the analysis of causes of death, reflecting several sources of medical information including medical reports, death certificates, information reported by the general practitioner and the family. The evaluation was conducted independently by senior cardiologists, with a comprehensive review of cases with an uncertain cause of death and additional analyses of randomised causes of death. If there were discrepancies between the observers or an inconclusive report, the cases were re-analysed to reach an agreement.

Then, the causes of death were grouped by major categories: infection, graft failure (including primary and late graft failure, cardiac allograft vasculopathy as well as rejection), sudden death, cardiovascular (including MACE), malignancy, other (including renal failure, haemorrhage, multi-organ failure and mechanical complications) and unknown causes using the following definitions:

- Graft failure was defined as death resulting from severe graft failure (cardiogenic shock, end-stage heart failure) due to either primary graft dysfunction and late graft dysfunction independently of the underlying causes (allograft rejection, cardiac allograft vasculopathy, restrictive physiology, unknown aetiology).

- SCD was defined according to the international definition.

- Cardio Cardiovascular deaths were defined as death due to major adverse cardiovascular events (MACE) including stroke, limb ischemia.

- Malignancy was defined as death related to progressive, end-stage cancer, escaping chemotherapy, often metastatic.

- Infection was defined as death related to a documented active infection, sepsis, septic shock.

- Other was defined as deaths due to renal failure, bleeding complications and surgical complications.

**Supplementary Figure 1. Cumulative incidence of causes of death in heart transplant recipients (*N* = 1220) within the first year. ***

*
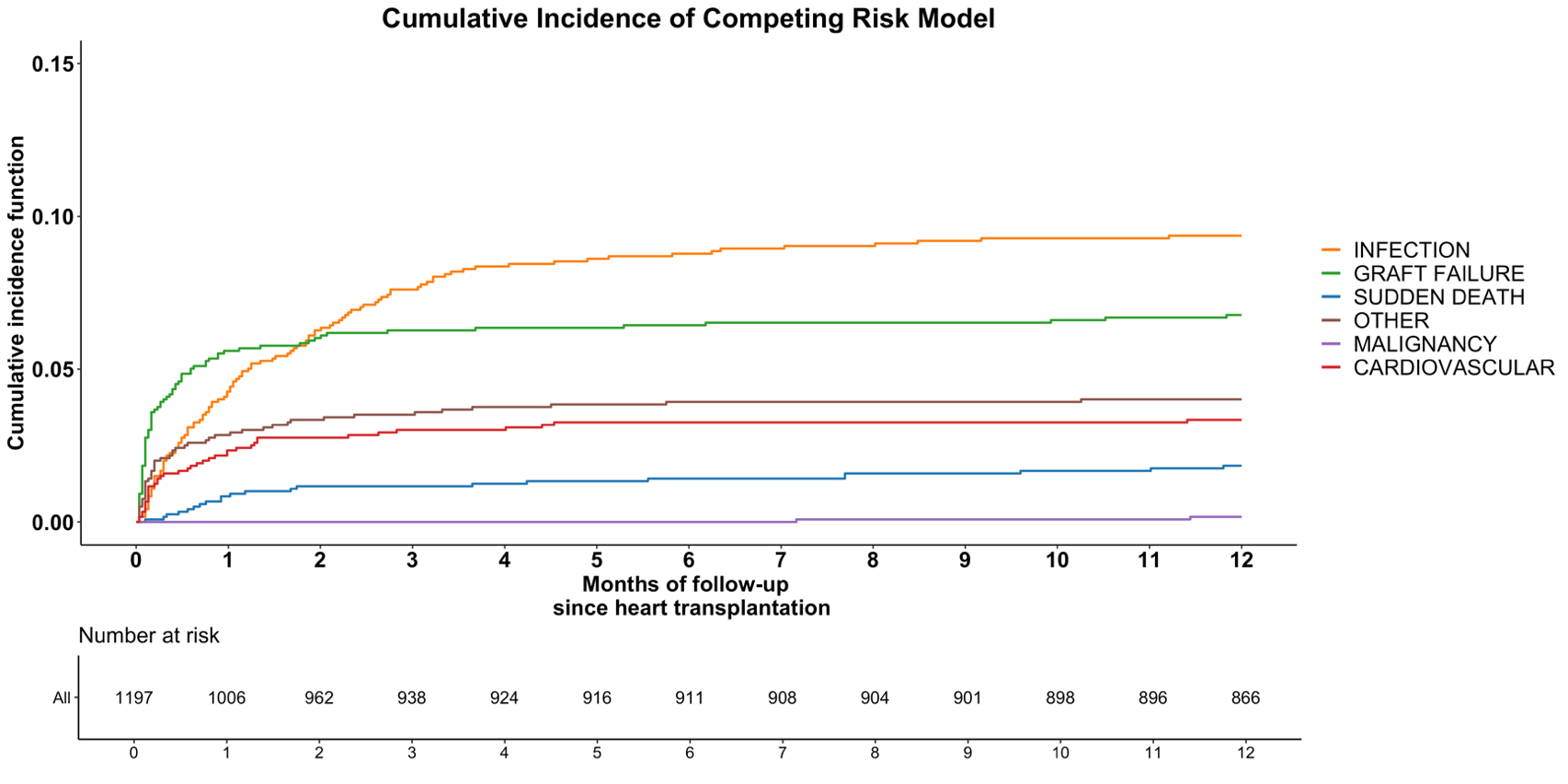
* Patients with an unknown cause of death have been excluded in the figure.*

**Supplementary Table 1: Characteristics of the overall cohort at the time of transplantation and among the 1-year survivors after transplantation.**

|  | | **Overall** | | | **One-year**  **survivors** | | | ***N*** | | | |  |  |
| --- | --- | --- | --- | --- | --- | --- | --- | --- | --- | --- | --- | --- | --- |
|  | | **(*N* = 1220)** | | | **(*N* = 905)** | | |  | | | |  |  |
| **Donor characteristics** | |  | | |  | | |  | |  |  |  |  |
| **Age** (years), mean (SD) | | | 45.6 (13.3) | | | 45.4 (13.2) | | | 1220 | |  |  |  |
| **Sex (men)**, *n* (%) | | | 783 (64.2%) | | | 597 (66.0%) | | | 1220 | |  |  |  |
| **Tobacco**, *n* (%) | | | 415 (46.4%) | | | 315 (45.8%) | | | 894 | |  |  |  |
| **Hypertension**, *n* (%) | | | 165 (18.4%) | | | 123 (17.9%) | | | 895 | |  |  |  |
| **Diabetes mellitus**, *n* (%) | | | 19 (3.12%) | | | 19 (3.13%) | | | 609 | |  |  |  |
| **Alcohol**, *n* (%) | | | 275 (30.7%) | | | 214 (31.1%) | | | 895 | |  |  |  |
| **BMI** (kg/m^2^), mean (SD) | | | 25.4 (4.7) | | | 25.4 (4.8) | | | 1211 | |  |  |  |
| **Creatinine clearance** (mL/min/1.73m^2^), mean (SD) | | | 99.5 (25.3) | | | 99.5 (25.4) | | | 609 | |  |  |  |
| **Recipient characteristics** | |  | | |  | | |  | | | | | |
| **Age** (years), mean (SD) | | 48.8 (13.1) | | | 47.9 (13.0) | | | 1220 | | | | |  |
| **Sex (men)**, *n* (%) | | 939 (77.0%) | | | 711 (78.6%) | | | 1220 | | | | |  |
| **BMI** (kg/m^2^), mean (SD) | | 24.4 (4.2) | | | 24.3 (4.2) | | | 1220 | | | | |  |
| **Non-Caucasian ethnicity**, *n* (%) | | 324 (26.6%) | | | 255 (28.2%) | | | 1220 | | | | |  |
| **Primary heart disease**, *n* (%) | |  | | |  | | | 1220 | | | | |  |
| Dilated cardiomyopathy | | 533 (43.7%) | | | 404 (44.6%) | | |  | | | | |  |
| Ischaemic cardiomyopathy | | 380 (31.1%) | | | 285 (31.5%) | | |  | | | | |  |
| Re-transplantation | | 26 (2.1%) | | | 21 (2.3%) | | |  | | | | |  |
| Congenital | | 67 (5.5%) | | | 44 (4.9%) | | |  | | | | |  |
| Other | | 214 (17.5%) | | | 151 (16.7%) | | |  | | | | |  |
| **Hypertension**, *n* (%) | | 356 (30.2%) | | | 282 (32.2%) | | | 1177 | | | | |  |
| **Diabetes mellitus**, *n* (%) | | 216 (17.8%) | | | 144 (16.0%) | | | 1216 | | | | |  |
| **History of smoking**, *n* (%) | | 595 (49.3%) | | | 475 (53.1%) | | | 1208 | | | | |  |
| **Long-term MCS**, *n* (%) | | 158 (13.0%) | | | 122 (13.5%) | | | 1220 | | | | |  |
| **ECMO at transplant**, *n* (%) | | 314 (25.7%) | | | 216 (23.9%) | | | 1220 | | | | |  |
| **Transplant baseline characteristics** | | | | |  | | |  | | | | |  |
| **Cold ischaemic time** (min), mean (SD) | | 188 (58.2) | | | 185 (58.2) | | | 1217 | | | | |  |
| **Induction therapy,** *n* (%) | |  | | |  | | | 1220 | | | | |  |
| ATG | | 1115(91.4%) | | | 834 (92.2%) | | |  | | | | |  |
| Others | | 20 (1.6%) | | | 10 (1.1%) | | |  | | | | |  |
| IL2-R inhibitor | | 85 (7.0%) | | | 61 (6.7%) | | |  | | | | |  |
| **Sex mismatch** (Df Rm), *n* (%) | | 266 (21.8%) | | | 192 (21.2%) | | | 1220 | | | | |  |
| **Combined transplantation**, *n* (%) | | 67 (5.5%) | | | 43 (4.8%) | | | 1220 | | | | |  |
| **CMV mismatch** (D+ R-), *n* (%) | | 221 (18.8%) | | | 171 (19.4%) | | | 1173 | | | | |  |
| **Immunology and histology** | |  | | |  | | |  | | | | |  |
| **HLA mismatch (A/B/DR)**, mean (SD) | | 5.0 (0.9) | | | 5.0 (0.9) | | | 1182 | | | | |  |
| **Pre-formed DSA**, *n* (%) | | 347 (29.4%) | | | 261 (29.0%) | | | 1179 | | | | |  |
| **TCMR at 1 year**, *n* (%) | | 443 (43.2%) | | | 424 (47.2%) | | | 1026 | | | | |  |
| **AMR at 1 year**, *n* (%) | | 82 (8.0%) | | | 75 (8.3%) | | | 1027 | | | | |  |

*AMR: antibody-mediated rejection; ATG: antithymocyte globulin; BMI: body mass index; CMV: cytomegalovirus; Df Rm: female donor and male recipient; DSA: donor-specific antibodies; ECMO: extracorporeal membrane oxygenation; HTx: heart transplantation; ID: immunodominant; IL2-r: interleukin-2 receptor; LVEF: left ventricular ejection fraction; MCS: mechanical circulatory support; MFI: mean fluorescence intensity; MMF: mycophenolate mofetil; SD: standard deviation; TCMR: T cell–mediated rejection.*

**Supplementary Table 2. Baseline characteristics of patients in relation to survival status and mode of death (N = 905)**

|  | **All patients** | **Other deaths** | **SCD** | **Survivors** | ***p*_overall_** | ***N*** |
| --- | --- | --- | --- | --- | --- | --- |
|  | **(*N* = 905)** | **(*N* = 163)** | **(*N* = 46)** | **(*N* = 696)** |  |  |
| **Donor characteristics** | |  |  |  |  |  |
| **Age** (years), mean (SD) | 45.4 (13.2) | 46.2 (12.8) | 49.3 (10.8) | 44.9 (13.4) | 0.061 | 905 |
| **Sex (men)**, *n* (%) | 597 (66.0%) | 112 (68.7%) | 33 (71.7%) | 452 (64.9%) | 0.460 | 905 |
| **Tobacco**, *n* (%) | 315 (45.8%) | 59 (47.2%) | 14 (45.2%) | 242 (45.5%) | 0.940 | 688 |
| **Hypertension**, *n* (%) | 123 (17.9%) | 19 (15.1%) | 4 (12.9%) | 100 (18.8%) | 0.472 | 689 |
| **Diabetes mellitus**, *n* (%) | 19 (3.13%) | 1 (1.20%) | 0 (0.00%) | 18 (3.59%) | 0.576 | 607 |
| **Alcohol consumption**, *n* (%) | 214 (31.1%) | 38 (30.2%) | 12 (38.7%) | 164 (30.8%) | 0.635 | 689 |
| **BMI** (kg/m^2^), mean (SD) | 25.4 (4.79) | 25.4 (4.25) | 24.6 (4.13) | 25.4 (4.95) | 0.504 | 902 |
| **eGFR** (mL/min/1.73m2), mean (SD) | 99.4 (25.4) | 98.1 (22.1) | 107 (19.2) | 99.3 (26.1) | 0.361 | 605 |
| **Chronic kidney failure**, *n* (%) | 181 (29.9%) | 32 (39.0%) | 4 (18.2%) | 145 (28.9%) | 0.086 | 605 |
| **Recipient characteristics** |  |  |  |  |  |  |
| **Age** (years), mean (SD) | 47.9 (13.0) | 50.9 (11.9) | 40.7 (14.2) | 47.7 (13.0) | <0.001 | 905 |
| **Sex (men)**, *n* (%) | 711 (78.6%) | 133 (81.6%) | 33 (71.7%) | 545 (78.3%) | 0.335 | 905 |
| **BMI** (kg/m^2^), mean (SD) | 24.3 (4.16) | 25.0 (4.34) | 24.8 (4.82) | 24.1 (4.05) | 0.056 | 905 |
| **Non-Caucasian ethnicity**, *n* (%) | 255 (28.2%) | 34 (20.9%) | 19 (41.3%) | 202 (29.0%) | 0.014 | 905 |
| **Hypertension**, *n* (%) | 282 (32.2%) | 65 (40.6%) | 15 (34.9%) | 202 (30.1%) | 0.034 | 875 |
| **Diabetes mellitus**, *n* (%) | 144 (16.0%) | 37 (22.7%) | 8 (17.4%) | 99 (14.3%) | 0.030 | 902 |
| **Tobacco**, *n* (%) | 475 (53.1%) | 98 (60.1%) | 20 (43.5%) | 357 (52.0%) | 0.073 | 895 |
| **Long-term MCS**, *n* (%) | 122 (13.5%) | 26 (16.0%) | 0 (0.00%) | 96 (13.8%) | 0.018 | 905 |
| **ECMO at transplant**, *n* (%) | 216 (23.9%) | 39 (23.9%) | 12 (26.1%) | 165 (23.7%) | 0.935 | 905 |
| **Transplant baseline characteristics** | |  |  |  |  |  |
| **Cold ischaemic time** (min), mean (SD) | 185 (58.2) | 185 (57.8) | 192 (53.8) | 184 (58.6) | 0.640 | 905 |
| **Sex mismatch** (Df Rm), *n* (%) | 192 (21.2%) | 33 (20.2%) | 8 (17.4%) | 151 (21.7%) | 0.744 | 905 |
| **Combined transplantation**, *n* (%) | 43 (4.75%) | 6 (3.68%) | 3 (6.52%) | 34 (4.89%) | 0.628 | 905 |
| **CMV mismatch** (D+ R-), *n* (%) | 171 (19.4%) | 44 (27.5%) | 5 (11.4%) | 122 (18.0%) | 0.009 | 883 |
| **Immunology and histology** | |  |  |  |  |  |
| **HLA mismatch (A/B/DR)**, mean (SD) | 4.99 (0.96) | 4.97 (0.98) | 5.20 (0.92) | 4.98 (0.95) | 0.328 | 880 |
| **Pre-formed DSA**, *n* (%) | 261 (29.0%) | 44 (27.7%) | 19 (41.3%) | 198 (28.5%) | 0.166 | 899 |
| **TCMR at 1 year**, *n* (%) | 424 (47.2%) | 85 (52.1%) | 24 (53.3%) | 315 (45.7%) | 0.230 | 898 |
| **AMR at 1 year**, *n* (%) | 75 (8.34%) | 11 (6.75%) | 6 (13.3%) | 58 (8.39%) | 0.369 | 899 |

*AMR: antibody-mediated rejection; ATG: antithymocyte globulin; BMI: body mass index; CMV: cytomegalovirus; Df Rm: female donor and male recipient; DSA: donor-specific antibodies; ECMO: extracorporeal membrane oxygenation; HTx: heart transplantation; ID: immunodominant; IL2-r: interleukin-2 receptor; LVEF: left ventricular ejection fraction; MCS: mechanical circulatory support; MFI: mean fluorescence intensity; MMF: mycophenolate mofetil; SD: standard deviation; TCMR: T cell–mediated rejection.*

**Supplementary Table 3. Annual incidence of sudden cardiac death in different subgroups.**

The incidence of sudden death for each sub-population group was defined by the presence of one of the risk factors. The annual incidence is represented per 1,000 person-years for all causes of death.

| **Subgroups** | **Annual incidence per 1,000 person-years (95% CI)** | |
| --- | --- | --- |
| Donor age ≥ 50 years | 17.5 (12.4-24.0) |  |
| Recipient age ≤ 50 years | 14.9 (10.7-20.1) |  |
| Pre-formed DSA | 22.6 (14.7-33.1) |  |
| Non-Caucasian ethnicity | 15.7 (9.6-24.2) |  |
| Last LVEF ≤ 55% | 24.0 (12.4-41.9) |  |
| Donor age ≥ 50 years & recipient age ≤ 50 years | 24.6 (15.6-37.0) |  |
| Donor age ≥ 50 years & pre-formed DSA | 34.4 (20.4-54.4) |  |
| Recipient age ≤ 50 years & pre-formed DSA | 26.0 (15.1-41.6) |  |
| Last LVEF ≤ 55% and pre-formed DSA | 44.0 (14.3-100.3) |  |

*DSA: donor-specific antibodies; LVEF: left ventricular ejection fraction*

**Supplementary Table 4. Multivariate analysis of competitive risks with antibody-mediated rejection (AMR) grade > 2 instead of pre-formed DSA.**

This table shows the association of clinical, immunological, functional and structural parameters associated with sudden cardiac death in the multivariate Cox analysis, with competitive risks taking into account all other causes of death except unknown causes.

| ***N* = 885** | **HR [95% CI]** | ***p*** |
| --- | --- | --- |
| **Donor characteristics** |  |  |
| **Age** (10-year increment) | 1.46 [1.14;1.85] | 0.003 |
| **Recipient characteristics** |  |  |
| **Age** (10-year increment) | 0.72 [0.58;0.89] | 0.002 |
| **Non-Caucasian ethnicity No** | 1 | - |
| **Yes** | 1.71 [0.94;3.13] | 0.078 |
| **Immunology** |  |  |
| **AMR grade ≥ 2 No** | 1 | - |
| **Yes** | 7.14 [2.15;23.73] | 0.001 |
| **Echocardiography parameters** |  |  |
| **Last LVEF** (10% increment) | 0.72 [0.55;0.96] | 0.025 |

*CI: confidence interval, presented as [lower limit;upper limit]; LVEF: left ventricular ejection fraction.*

**Supplementary Table 5. Factors associated with sudden cardiac death in the multivariate Cox analysis with the competitive risk model.**

This table shows the association of clinical, immunological, functional and structural parameters associated with sudden cardiac death in the multivariate Cox analysis, with competitive risks taking into account all other causes of death including unknown causes.

| ***N* = 885** | **HR [95% CI]** | ***p*** |
| --- | --- | --- |
| **Donor characteristics** |  |  |
| **Age** (10-year increment) | 1.44 [1.14;1.82] | 0.003 |
| **Recipient characteristics** |  |  |
| **Age** (10-year increment) | 0.69 [0.57;0.85] | <0.001 |
| **Non-Caucasian ethnicity No** | 1 | - |
| **Yes** | 1.84 [1.02;3.31] | 0.042 |
| **Immunology** |  |  |
| **Pre-formed DSA No** | 1 | - |
| **Yes** | 2.17 [1.17;4.04] | 0.014 |
| **Echocardiography parameters** |  |  |
| **Last LVEF** (10% increment) | 0.80 [0.62;1.03] | 0.086 |

*CI: confidence interval, presented as [lower limit;upper limit]; AMR: antibody-mediated rejection; LVEF: left ventricular ejection fraction.*

**Supplementary Table 6. Factors associated with sudden cardiac death in the multivariate Cox analysis stratified by centre.**

This table shows the association of clinical, immunological, functional and structural parameters associated with sudden cardiac death in the multivariate Cox analysis, stratified by centre.

| ***N* = 885** | **HR [95% CI]** | ***p*** |
| --- | --- | --- |
| **Donor characteristics** |  |  |
| **Age** (10-year increment) | 1.45 [1.10;1.90] | 0.008 |
| **Recipient characteristics** |  |  |
| **Age** (10-year increment) | 0.73 [0.59;0.90] | 0.003 |
| **Non-Caucasian ethnicity No** | 1 | - |
| **Yes** | 1.83 [1.00;3.37] | 0.051 |
| **Immunology** |  |  |
| **Pre-formed DSA No** | 1 | - |
| **Yes** | 2.48 [1.33;4.61] | 0.004 |
| **Echocardiography parameters** |  |  |
| **Last LVEF** (10% increment) | 0.75 [0.55;1.00] | 0.052 |

*CI: confidence interval, presented as [lower limit;upper limit]; AMR: antibody-mediated rejection; LVEF: left ventricular ejection fraction.*

**Supplementary References**

1. Berry GJ, Burke MM, Andersen C, et al. The 2013 international society for heart and lung transplantation working formulation for the standardization of nomenclature in the pathologic diagnosis of antibody-mediated rejection in heart transplantation. J Heart Lung Transplant 2013;32(12):1147-62.

2. Stewart S, Winters GL, Fishbein MC, et al. Revision of the 1990 working formulation for the standardization of nomenclature in the diagnosis of heart rejection. J Heart Lung Transplant 2005; 24(11):1710-20.

3. Lang RM, Badano LP, Victor MA, et al. Recommendations for cardiac chamber quantification by echocardiography in adults: an update from the American Society of Echocardiography and the European Association of Cardiovascular Imaging. J Am Soc Echocardiogr 2015; 28: 1-39.e14.

4. Loupy A, Vernerey D, Viglietti D, et al. Determinants and outcomes of accelerated arteriosclerosis: major impact of circulating antibodies. Circ Res 2015;117(5):470-82.

5. ﻿Kobashigawa J., Crespo-Leiro MG., Ensminger SM., et al. ISHLT CONSENSUS Report from a consensus conference on antibody-mediated rejection in heart transplantation. J Heart Lung Transplant 2011. doi:10.1016/j.healun.2010.11.003.

6. Mehra MR, Crespo-Leiro MG, Dipchand A, et al. International Society for Heart and Lung Transplantation working formulation of a standardized nomenclature for cardiac allograft vasculopathy, 2010. J Heart Lung Transplant 2010;29(7):717-27

7. ﻿Coutance G, D’Orio V, Belin L, et al. Favorable outcome of an exclusively posttransplant prophylactic strategy after heart transplantation in recipients with high immunological risk. Transplantation. 2019;103(7):1439-1449. doi:10.1097/TP.0000000000002503

8. ﻿Loupy A, Loupy A, Coutance G, et al. Identification and characterization of trajectories of cardiac allograft vasculopathy after heart transplantation: A population-based study. Circulation. 2020;141(24):1954-1967. doi:10.1161/CIRCULATIONAHA.119.044924.

9. Patel JK, Coutance G, Loupy A, et al. Complement inhibition for prevention of antibody-mediated rejection in immunologically high-risk heart allograft recipients. Am J Transplant. 2021;21(7):2479-2488. doi:10.1111/ajt.1642010.

10. Khush KK, Cherikh WS, Chambers DC, *et al.* The International Thoracic Organ Transplant Registry of the International Society for Heart and Lung Transplantation: thirty-sixth adult heart transplantation report – 2019; focus theme: donor and recipient size match. J Heart Lung Transplant 2019; 38: 1056-66.
